# Features of the Opportunistic Behaviour of the Marine Bacterium *Marinobacter algicola* in the Microalga *Ostreococcus tauri* Phycosphere

**DOI:** 10.3390/microorganisms9081777

**Published:** 2021-08-20

**Authors:** Jordan Pinto, Raphaël Lami, Marc Krasovec, Régis Grimaud, Laurent Urios, Josselin Lupette, Marie-Line Escande, Frédéric Sanchez, Laurent Intertaglia, Nigel Grimsley, Gwenaël Piganeau, Sophie Sanchez-Brosseau

**Affiliations:** 1 Sorbonne Université, CNRS, UMR 7232 Biologie Intégrative des Organismes Marins (BIOM), Observatoire Océanologique, 66650 Banyuls-sur-Mer, France; marc.krasovec@obs-banyuls.fr (M.K.); lupettejosselin@yahoo.fr (J.L.); frederic.sanchez@obs-banyuls.fr (F.S.); nigel.grimsley@obs-banyuls.fr (N.G.); gwenael.piganeau@obs-banyuls.fr (G.P.); 2 Sorbonne Université, CNRS, USR 3579 Laboratoire de Biodiversité et Biotechnologies Microbiennes (LBBM), Observatoire Océanologique, 66650 Banyuls-sur-Mer, France; raphael.lami@obs-banyuls.fr; 3 Department of Plant Sciences, University of Oxford, Oxford OX1 3RB, UK; 4 Université de Pau et des Pays de l’Adour, E2S UPPA, CNRS, IPREM, 64000 Pau, France; regis.grimaud@univ-pau.fr (R.G.); laurent.urios@univ-pau.fr (L.U.); 5 Université de Bordeaux, CNRS, UMR 5200 Laboratoire de Biogenèse Membranaire, 33140 Villenave d’Ornon, France; 6 Sorbonne Université, CNRS, FR 3724, Observatoire Océanologique, 66650 Banyuls-sur-Mer, France; marie-line.escande@obs-banyuls.fr (M.-L.E.); laurent.intertaglia@obs-banyuls.fr (L.I.)

**Keywords:** *Marinobacter*, bacteria, *Ostreococcus*, phycosphere, phytoplankton, cocultures

## Abstract

Although interactions between microalgae and bacteria are observed in both natural environment and the laboratory, the modalities of coexistence of bacteria inside microalgae phycospheres in laboratory cultures are mostly unknown. Here, we focused on well-controlled cultures of the model green picoalga *Ostreococcus tauri* and the most abundant member of its phycosphere, *Marinobacter algicola*. The prevalence of *M. algicola* in *O. tauri* cultures raises questions about how this bacterium maintains itself under laboratory conditions in the microalga culture. The results showed that *M. algicola* did not promote *O. tauri* growth in the absence of vitamin B_12_ while *M. algicola* depended on *O. tauri* to grow in synthetic medium, most likely to obtain organic carbon sources provided by the microalgae. *M. algicola* grew on a range of lipids, including triacylglycerols that are known to be produced by *O. tauri* in culture during abiotic stress. Genomic screening revealed the absence of genes of two particular modes of quorum-sensing in *Marinobacter* genomes which refutes the idea that these bacterial communication systems operate in this genus. To date, the ‘opportunistic’ behaviour of *M. algicola* in the laboratory is limited to several phytoplanktonic species including Chlorophyta such as *O. tauri*. This would indicate a preferential occurrence of *M. algicola* in association with these specific microalgae under optimum laboratory conditions.

## 1. Introduction

Phytoplankton and bacterial dynamics are closely linked in coastal marine environments [1,2]. Numerous findings suggest that these specific relationships result from a long coexistence in the oceans for more than 200 million years [3,4,5]. Some bacterial strains are frequently isolated from natural blooms and cultivated in the laboratory [6,7,8]. Phytoplankton exudates can be important carbon substrates for bacteria, especially in early phytoplankton bloom conditions [9,10] although other carbon sources might also be important for bacterial growth [9]. In turn, it is clear now that heterotrophic bacteria can improve or inhibit microalgal growth [10,11,12,13]. Some bacteria provide marine algae with essential vitamins and nutrients while others compete with microalgae for nutrients or produce algicidal components [10], but very often the compounds involved in the partnership have not been fully characterised [11]. In addition, the coexistence of many bacteria in microalgae laboratory cultures is still not completely understood [12].

*Ostreococcus* is a widely distributed marine phytoplankton genus detected around the world [13] and contributes significantly to primary production in oligotrophic waters. *Ostreococcus tauri* was discovered in the Mediterranean Thau Lagoon in 1994 and was described as the smallest free-living eukaryotic cell [14]. This species can be observed as a dominant species during phytoplanktonic blooms in coastal seas [15]. *O. tauri* picoplanktonic cells (<2 µm) are auxotrophic for vitamin B_1_ (thiamine) and vitamin B_12_ (cobalamin). They depend on exogenous sources of vitamin or vitamin precursors for growth [16,17,18,19,20]. *O. tauri* possesses the *metH* gene coding for vitamin B_12_ methionine synthase but lacks the *metE* gene coding for B_12_-independent methionine synthase which allows growth in the absence of vitamin B_12_ [21]. It has been previously demonstrated that in the absence of vitamin B_12_, diatoms lacking the functional *metE* gene cannot survive [22]. In B_12_ depleted environments, microalgae which lack methionine synthase activity would need available sources of B_12_ produced exclusively by prokaryotes in the environment [23]. The biosynthetic pathway of vitamin B_12_, a complex tetrapyrrole, appears to be confined to prokaryotes [23]. It is intriguing that many oligotrophic species such as *O. tauri* are nonetheless vitamin auxotrophs [20,24] and so must be able to obtain a ready source of these organic micronutrients from their surroundings. Specific biotic interactions between algae and bacteria have been shown to take place for B_12_ acquisition to the algae from the bacteria [18,19] that in exchange benefit from algal exudates [18,22]. Recent studies have demonstrated that heterotrophic bacteria can satisfy microalgal requirements for B_12_ via mutualistic interactions [6,18]. It has been shown recently that cross-exchange of B-vitamins underpins a mutualistic interaction between *O. tauri* and the alpha-proteobacterium *Dinoroseobacter shiba* [20]. To survive in oligotrophic environments depleted in cobalamin, we expect that *O. tauri* would take up cobalamin produced by bacteria.

The bacterial diversity of the phycosphere of the green algae *Ostreococcus tauri* RCC4221 cultivated in the laboratory revealed a large dominance of *M. algicola* [25], hereafter noticed as *M. algicola* OT. The *M. algicola*-type strain was isolated from a dinoflagellate culture and was then frequently detected in dinoflagellate and coccolithophorid cultures [26,27]. Bacteria from the *Marinobacter* genus are found in numerous marine habitats including the deep ocean, costal seawater, sediments and sea-ice, and in associated animal or algal hosts. Many *Marinobacter* strains can adopt two life-styles, living planktonically or as biofilms [28]. *Marinobacter* species are frequently present in diverse microalgae phycospheres in laboratory cultures. Strains of *M. adhaerens* are frequently detected in diatom cultures [26,27,29,30]. They interact with diatom cells and are involved in the aggregation of microalgae [27,31]. An important feature is the high prevalence of *M. algicola* OT in *O. tauri* cultures [26] and it raises the question of how these bacteria could communicate to rapidly grow and spread in their environment. Relatedly, colonisation of the phycosphere by bacterial populations can occur through quorum sensing (QS) [32]. QS is a widespread cell to cell bacterial communication method that regulates and synchronises numerous bacterial activities through the diffusion of autoinducer (AI) molecules [33]. However, to date, only one study investigating QS in *Marinobacter* has been previously published [34].

In this work, we explored several hypotheses to investigate whether any positive, neutral or negative interaction occurred between the algae *O. tauri* RCC4221 and the bacterial strain *M. algicola* OT. Since *O. tauri* was auxotrophic for vitamin B_12_, the possibility that *M. algicola* provided the vitamin to the algae was tested. After *M. algicola* OT was isolated from a culture of *O. tauri* RCC4221 and maintained under laboratory conditions, coculture experiments with inoculation of different *M. algicola* OT concentrations were conducted to check whether *O. tauri* RCC4221 needs *M. algicola* OT for its growth under different conditions. We combined the searches in genomes for genes coding for proteins possibly involved in such interactions with experiments aimed at supporting the genomic data. Then, given the ability of *Marinobacter* species to degrade lipids [31] and the diversity of fatty acids and triacylglycerols (TAGs) produced by *O. tauri* [35], the capacity of *M. algicola* OT to use lipids for growth was tested. We again developed a double approach to combine bioinformatics screening of *Marinobacter* genomes and experiments on *M. algicola* OT to find insights of QS capacity in *Marinobacter*. Lastly, a phylogenetic reconstruction including the *M. algicola* strain from the *O. tauri* RCC4221 culture and all available *Marinobacter* 16S sequences was first performed to detect whether there was any evidence of a common evolutionary trend between *Marinobacter* strains found in microalgae cultures and if they share similar phylogenetic traits.

## 2. Material and Methods

### 2.1. Phylogenetic Analysis

**Figure 1:**
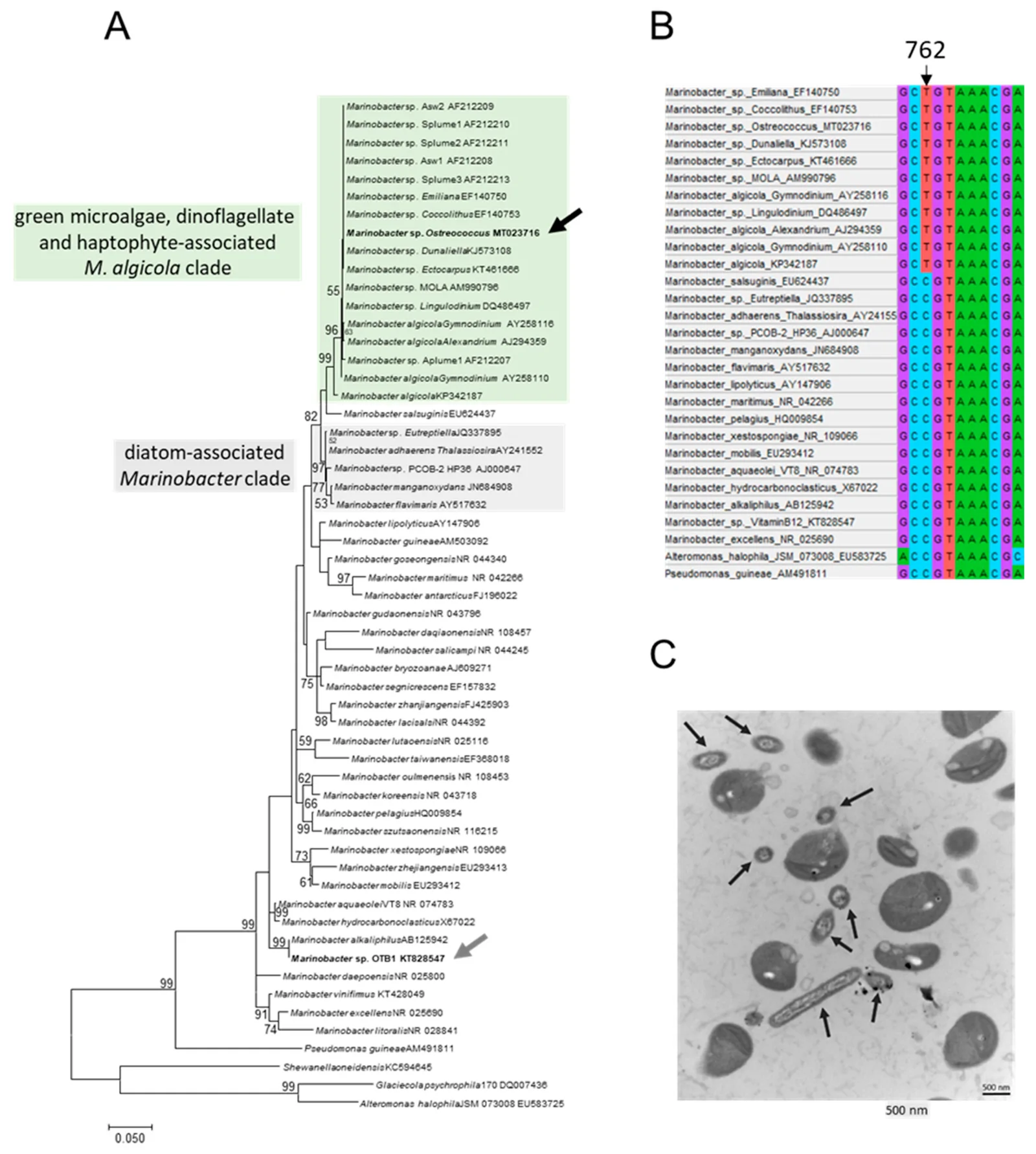
Phylogenetic and morphological features of *M. algicola* OT MT023716. (**A**) ML tree from a 1415-position 16S alignment analysed with GTR + G + I model and 100 bootstrap replicates. These 56 nucleotide sequences constitute the totality of *Marinobacter* species. The *M. algicola* OT MT023716 sequence (black arrow) obtained from the *O. tauri* RCC4221 culture (in the ‘green microalgae, dinoflagellate and haptophyte-associated *M. algicola* clade’), the sequence from the unique *Marinobacter* strain previously tested for AI production (in the ‘diatom-associated *Marinobacter* clade’) and the OTB1 sequence from a *Marinobacter* strain previously observed in another *O. tauri* strain (RCC745) (grey arrow) are shown in bold. Branch lengths are proportional to the number of substitutions per site. (**B**) Partial alignment showing position 762 with the T nucleotide shared by sequences from only the *M. algicola* clade. (**C**) Transmission electron micrograph of *O. tauri* culture supernatant cells showing *O. tauri* cells (large cells approximately 0.8 µm in size) and rod-shaped bacteria (black arrows) with size characteristics corresponding to *M. algicola* DG893 [26]. Black arrows show bacterial cells in different plane slices (probably *M. algicola*).

To comment on the phylogenetic position of our *M. algicola* OT strain with respect to the other strains, a molecular phylogeny of 16S rRNA gene sequences was performed using sequences from different *Marinobacter* species. The dataset comprises 56 nucleotide sequences from GenBank including the *M. algicola* OT sequence (MT023716), for a total of 1415 positions after multiple alignment using the ClustalW algorithm in MEGA X version 10.1.7 software (available at https://www.megasoftware.net/, accessed on 9 August 2021) [36]. A tree was constructed using a GTR + I (invariable sites proportion) + G (discrete gamma distribution) model selected using the ‘Find Best DNA Models’ option in MEGA X version 10.1.7 software (available at https://www.megasoftware.net/, accessed on 9 August 2021) [36] and the maximum likelihood (ML) method using 100 bootstrap replicates (BP). All accession numbers are indicated in the tree (Figure 1). Particular attention was focused on the *M. algicola* clade with a comparison of sequences and the identification of specifically shared nucleic acid characteristics.

### 2.2. Transmission Electron Microscopy

We used transmission electron microscopy as routinely performed in the laboratory [37], to check the possibility of a close physical interaction between microalgae and bacteria. A pellet of five hundred million microalgae cells in exponential growth phase was prefixed in L1 culture medium with glutaraldehyde 1% (#16320, Electron Microscopy Sciences, Hatfield, PA, USA) for 30 min at room temperature. Then, the cells were centrifuged at 2500× *g* (Beckman Avanti™ centrifuge J-20XP, Beckman Coulter, Fullerton, CA, USA) for 25 min at 8 °C. Pellets were then resuspended in 40 µL of 1% low melting point agar (#A9414, Sigma, St. Louis, MO, USA) in L1 culture medium. After solidification, the plug was fixed for 2 h at 4 °C with 2.5% glutaraldehyde in one volume of 0.4 M sodium cacodylate buffer (#12300, Electron Microscopy Sciences, Hatfield, PA, USA) and two volumes of culture medium. The plug was then washed three times for 15 min in 0.2 M sodium cacodylate buffer, postfixed in 2% osmium tetroxide (#19150, Electron Microscopy Sciences, Hatfield, PA, USA) for 1 h and finally washed three times again as previously described. Samples were dehydrated in successive baths with increasing ethanol concentrations (70%, 95% and 100%) and propylene oxide (#O4332, Fisher Scientifics, Hampton, NH, USA). Samples were progressively impregnated and embedded in Epon 812 (#14120, Electron Microscopy Sciences, Hatfield, PA, USA). Thin sections were prepared with an ultramicrotome (Leica Ultracut R, Leica Biosystems, Nussloch, Germany) and stained with 2% uranyl acetate in 50% ethanol (ETOH) solution and 0.5% lead citrate in aqueous solution before examination on a transmission electron microscope (Hitachi H-7500, Hitachi, Tokyo, Japan).

### 2.3. O. tauri RCC4221 and M. algicola OT Cultures and Growth

#### 2.3.1. *O. tauri* RCC4221 Culture

*Ostreococcus tauri* strain RCC4221 was isolated in 1994 from the North-West MediterraneanThau lagoon [14] and maintained in the laboratory (cultures and cryopreservation). The *O. tauri* RCC4221 strain was grown in liquid medium in 50 mL aerated flasks (Sarstedt) at 20 °C under white light at approximately 100 µmol photons·m^−2^·s^−1^ using an LD 12:12 photoperiod. Cell growth was followed by measuring the cell concentrations in small aliquots (50 µL) of culture every two or three days with a flow cytometer (CytoFlex, Beckman Coulter, Fullerton, CA, USA). *O. tauri* cells were detected using the red fluorescence emission (FL3 acquisition at 670 nm) of chlorophyll pigments.

#### 2.3.2. *M. algicola* OT Culture

The *M. algicola* OT strain, used for the experiments in this study, is the same that the one initially isolated from *O. tauri* RCC4221 in 2016 [25]. More precisely, numerous *M. algicola* OTUs with partial 16S sequence (obtained by Illumina sequencing) were evidenced in this previous study [25]. After cultivation of the *O. tauri* RCC4221 phycosphere in solid medium, only one *M. algicola* strain could be successfully isolated, the *M. algicola* OT strain used in this study. Cell growth was followed either by optical density (OD) or by measuring the cell concentrations with a flow cytometer as above. In this latter case, for enumeration of bacteria, small aliquots (50 µL) of culture were used for measurement by flow cytometry every two or three days. Bacterial nucleic acids were labelled with SYBR^®^ Green I (#50513, Lonza, Basel, Switzerland) and detected by green fluorescence (FL1 acquisition at 530 nm) [38].

#### 2.3.3. Culture Media

For all *O. tauri* cultures and coculture experiments with *M. algicola* OT hereafter, ESAW-F/2 culture media [39] were prepared with artificial seawater and supplemented with nitrogen (NaNO_3_, 8.8 × 10^−5^ mol·L^−1^), phosphorus (NaH_2_PO_4_, 3.62 × 10^−5^ mol·L^−1^), vitamins (B_1_, 2.96 × 10^−7^ mol·L^−1^, B_12_, 1.48 × 10^−9^ mol·L^−1^ and H, 4.09 × 10^−9^ mol·L^−1^) and trace metals. A culture medium without vitamin B_12_ was also prepared for B_12_ depletion experiments. In addition, one growth experiment of *M. algicola* OT monoculture (positive control) was performed using a highly enriched medium, the marine broth (MB 2216) medium, typically used for bacteria growth.

### 2.4. O. tauri RCC4221 and M. algicola OT Coculture Experiments

*O. tauri* RCC4221 cultures used for coculture experiments were not axenic despite the use of antibiotic treatments. At the lowest possible bacterial contamination (approximately 3%) at the stationary phase, the *O. tauri* RCC4221 culture was diluted to 10^6^ cells·mL^−1^ in ESAW-F/2 medium and distributed into several 50 mL sterile flasks. To test the effects of *M. algicola* OT on *O. tauri* RCC4221, different final bacterial concentrations (5 × 10^6^ and 2 × 10^7^ *M. algicola* OT cells·mL^−1^) were added to the *O. tauri* culture RCC4221. A control (without the addition of *M. algicola* OT to the *O. tauri* RCC4221 culture) allowed us to visualise the growth pattern of the few remaining bacteria (fewer than 3 × 10^5^ cells·mL^−1^) after axenisation was not totally efficient. *M. algicola* OT cells were distinguished easily from other bacterial communities in *O. tauri* RCC4221 cultures owing to the fluorescence signal for *M. algicola* OT after SYBR Green labelling being lower than those for other bacteria. The same experiment was carried out in a medium without vitamin B_12_. As before, different final concentrations (5 × 10^6^ and 2 × 10^7^ *M. algicola* OT cells·mL^−1^) were added to the *O. tauri* culture. All coculture experiments were carried out in triplicates experiments and were performed for a total duration of 30 days.

### 2.5. M. algicola OT Growth Tests on Lipids

*M. algicola* OT (MT023716) was tested for its capacity to grow on palmitic acid (16:0), tricaprylin (TAG 8:0/8:0), tripalmitin (TAG 16:0/16:0/16:0), triolein (TAG 18:1/18:1/18:1), triarachidin (TAG 20:0/20:0/20:0) and hexadecyl palmitate (wax ester). Lipids were from Tokyo Chemical Industry Co. LTD, Tokyo, Japan. The strain was grown in marine broth (MB) overnight at 30 °C and resuspended at an optical density at 600 nm (OD_600nm_) of 0.1 in MB minimum [40]. Substrates were added at 0.1% (*w*/*v*) to the cell suspension in glass tubes and incubated at 30 °C under agitation at 150 rpm. Growth was monitored at 20, 44, 68 and 164 h by reading the OD_600nm_.

### 2.6. In Silico Genomic Screening of O. tauri Phycosphere Sequences and Complete Marinobacter Genomes

Whole-genome sequencing data of 19 *O. tauri* mutation accumulation line sets were used for genomic screening. These 19 *O. tauri* non-axenic culture lines (containing *O. tauri* and all bacterial communities from its phycosphere) were the result of a previous extinction-dilution experiment of 378 days, which corresponded to approximately 512 generations per line with 27 points of extinction dilution to one cell [41]. These lines were shown to exclusively exhibit *M. algicola* cultivable species at the end point [25]. To detect whether the genomes of the remaining bacteria present in the 19 non-axenic *O. tauri* line cultures possessed genes of interest, we aligned the raw reads against the target gene sequences (detailed in the following sections) using Burrows-Wheeler Aligner (BWA, http://bio-bwa.sourceforge.net, accessed on 26 July 2018) with standard parameters [42] and treated the resulting bam files with SAMtools (http://www.htslib.org, accessed on 26 July 2018) to obtain the coverage of these genes [43].

We performed BLASTP of translated sequences of interest against the reference proteomes of the 21 available and complete reference genomes and proteomes of *Marinobacter* species, including the *M. algicola* DG893 strain, the closest strain to date to *M. algicola* OT, which were also used for screening (all accession numbers are noticed in Table 2).

#### 2.6.1. Searching for Methionine Synthases Genes

We searched for evidence of methionine synthesis by *M. algicola* by screening the different genomic datasets. We focused on the *metH* methionine synthase gene (locus_tag = ‘MDG893_RS02235’, NZ_ABCP01000002) and the *metE* methionine synthase gene (locus_tag = ‘MDG893_RS14865’, NZ_ABCP01000027) identified from the *M. algicola* DG893 annotated genome (PRJNA19321).

#### 2.6.2. Searching for Lipid Degradation Genes

We searched evidence for lipid degradation genes. We focused on 2 genes already identified as important for lipid degradation [31]: fadB (Fatty acid degradation alpha subunit, TIGR02437) and fadA (3-ketoacyl-CoA thiolase, TIGR02445) from the fatty oxidation complex.

#### 2.6.3. Searching for Quorum-Sensing Genes

We sought evidence of QS-associated genes in the different databases. We focused on the reference QS genes from *Vibrio* species including *luxI* (AY275714) from *V. fischeri* [44], *luxR* (DQ108980) from *V. harveyi* [45], *luxS* (AY391122) from *V. alginolyticus*, *luxM* (DQ987706) from *V. splendidus* [46], *vanM* (KT258634) and *ainS* (AY277634) from *V. fischeri*.

### 2.7. In Silico Protein Analysis

In addition to genomic analysis, the presence of potential AI synthase and LuxR-like receptors in the species *M. algicola* was examined in the InterPro Database v69.0 (https://www.ebi.ac.uk/interpro/, accessed on 9 August 2021) [47] with the queries ‘IPR001690—autoinducer synthase’, ‘IPR000792—Transcription regulator LuxR, C-terminal’ and ‘IPR005143 Transcription factor LuxR-like, autoinducer-binding domain’. Once protein sequences were retrieved, protein domains were then identified using SMART 7 software (online resource, http://smart.embl.de/, accessed on 9 August 2021) [48] and aligned using the ClustalW algorithm under MEGA X version 10.1.7 software (available at https://www.megasoftware.net/, accessed on 9 August 2021) [36] with *Vibrio fischeri* LuxR and *Agrobacterium* TraR sequences to check for potential conserved amino-acids. A prediction of ligand binding on LuxR proteins was performed to test whether the identified LuxR proteins of *M. algicola* could effectively be involved in QS. The ligands were identified using COACH-D software (online resource, https://yanglab.nankai.edu.cn/COACH-D/, accessed on 9 August 2021) available in the I-TASSER version 5.1 package (online resource, https://zhanglab.dcmb.med.umich.edu/I-TASSER/download/, accessed on 9 August 2021) [49].

### 2.8. Evaluation of M. algicola OT Quorum-Sensing Capacities

We investigated in this study AI-1 (AHL-driven) and AI-2 (Furanosyl diester borate-driven) modes of QS. Functional experiments with different types of biosensors were conducted to evaluate the QS capacities of *M. algicola* OT through AI-1 and AI-2 production, using protocols based on previously published research conducted in our group [50]. Briefly, *M. algicola* OT culture supernatants were tested using *Pseudomonas putida* and *Escherichia coli*-based biosensors. *P. putida* F117 (pRK-C12; Kmr; ppuI:mpt) was used to detect long-chain (>8 carbons in the acyl side chain) N-acyl homoserine lactones (AHLs) [51,52,53], and *E. coli* MT102 (pJBA132) was used to evaluate the emission of short-chain (≤8 carbons in the acyl side chain) AHLs [53,54]. A mutant strain of *Vibrio harveyi*, BB170, was cultivated to detect the presence of AI-2 in the culture supernatants [55,56]. Protocols are precisely described in Text S1 (Supplementary Materials).

## 3. Results

### 3.1. Phylogenetic Position of M. algicola OT in the Global Marinobacter Species Tree

A phylogenetic reconstruction was performed to more precisely describe the *M. algicola* OT phylogenetic position and to determine if all *Marinobacter* strains found in phytoplankton cultures tend to form a cluster. *M. algicola* OT, isolated from a culture of *O. tauri* RCC4221 [25], belongs to the *M. algicola* phylogenetic group (BP = 99) (black arrow, Figure 1A). This clade also includes *M. algicola* strains from the dinoflagellates *Alexandrium* AJ294359, *Gymnodinium* AY258116, AY258110 and *Lingulodinium* DQ486497, the chlorophytes *Ostreococcus tauri* and *Dunaliella* KJ573108 and the haptophytes *Emiliana* EF140750 and *Coccolithus* EF140753. All sequences from this clade share a diagnostic nucleotide: a T is present at position 762 in all sequences (Figure 1B) while in all other sequences, a C is present at this position. While all sequences belonging to this clade share 97.4% sequence identity (1378 identical nucleotide sites over the 1415 base pairs in the total alignment), it is not possible to state if *M. algicola* strains found in dinoflagellate cultures are more closely related to each other than to the other strains given the relatively low variability level inside the clade. Transmission electron microscopy observations of *O. tauri* RCC4221 cultures revealed numerous bacterial cells that exhibited a morphology similar to *M. algicola* DG893 (Figure 1C) [56]. The bacterial cells and the microalgae did not physically interact (Figure 1C).

### 3.2. Phylogenetic Features of M. algicola Relative to Other Marinobacter Species Found in Phytoplankton Cultures

The closest phylogenetically related *Marinobacter* species to *M. algicola* is *M. salsuginis* which appeared as a sister group of *M. algicola* (BP = 47, considered weak support, Figure 1A) with two diagnostic characters: A at position 618 (instead of the G in other sequences) and T at position 688 (instead of the C in other sequences) (personnal observation). Other *Marinobacter* strains observed in phytoplanktonic cultures, such as *M. adhaerens* known to be associated with diatoms, do not belong to the *M. algicola* and *M. salsuginis* clades (Figure 1A). *Marinobacter* sp. OTB1 (grey arrow, Figure 1A), did not cluster in the *M. algicola* clade but appeared to be related to a more distant lineage (*M. alkaliphilus*). The 16S sequences from *Marinobacter* sp. OTB1 and *M. algicola* OT strains exhibited 44 differences over the 829 homologous sites, representing 5.3% of the differences over these partial 16S sequences, which made these two strains evolutionarily distant from each other.

### 3.3. Effect of M. algicola OT on O. tauri RCC4221 Growth in the Absence of Vitamin B_12_

**Figure 2:**
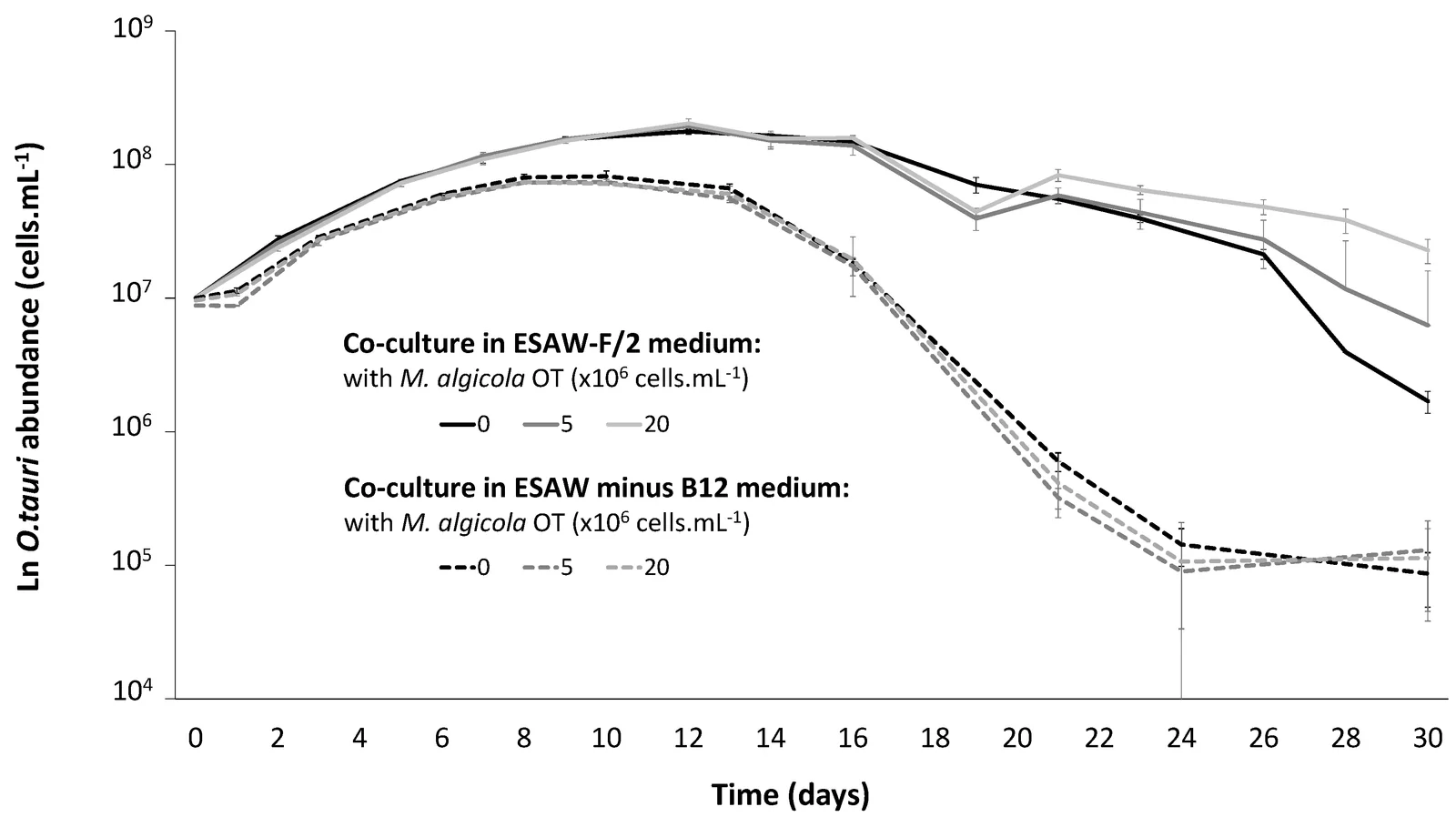
*O. tauri* RCC4221 growth in coculture with *M. algicola* OT MT023716. Cocultures were performed in complete ESAW- F/2 medium (solid curves) and in ESAW medium depleted in vitamin B_12_ (ESAW minus B_12_) medium (dotted curves).

Coculture experiments were compared after inoculation of *M. algicola* OT at different concentrations and in presence and absence of vitamin B_12_. The absence of vitamin B_12_ in the culture medium affected the growth curve of *O. tauri* RCC4221. In the presence of vitamin B_12_ (complete ESAW-F/2 medium), the maximal cell abundance was 2.5-fold higher than that in the vitamin B_12_-depleted medium. Moreover, the decay phase was much faster in the absence of vitamin B_12_ (Figure 2). Interestingly, when *M. algicola* OT was added to the cultures (at 5 × 10^6^ and 2 × 10^7^ cells·mL^−1^ in ESAW-F/2 complete medium), *O. tauri* was maintained at the highest density from day 26 to the end of the experiment (Figure 2). However, the presence of *M. algicola* OT in the culture of *O. tauri* RCC4221 in vitamin B_12_-depleted medium did not restore the growth pattern of the algae observed in the presence of vitamin B_12_. As an evidence of the bacterial capacity to synthesise methionine (if genes are transcribed and translated), we retrieved that *metH* and *metE* methionine synthase genes from several *O. tauri* mutation accumulation lines containing reads from bacterial communities from its phycosphere (Table 1) and also from in the genome of *M. algicola* DG893, the closest sequenced strain to *M. algicola* OT (Table 2).

### 3.4. M. algicola OT Growth Behaviour in Coculture with O. tauri RCC4221

**Figure 3:**
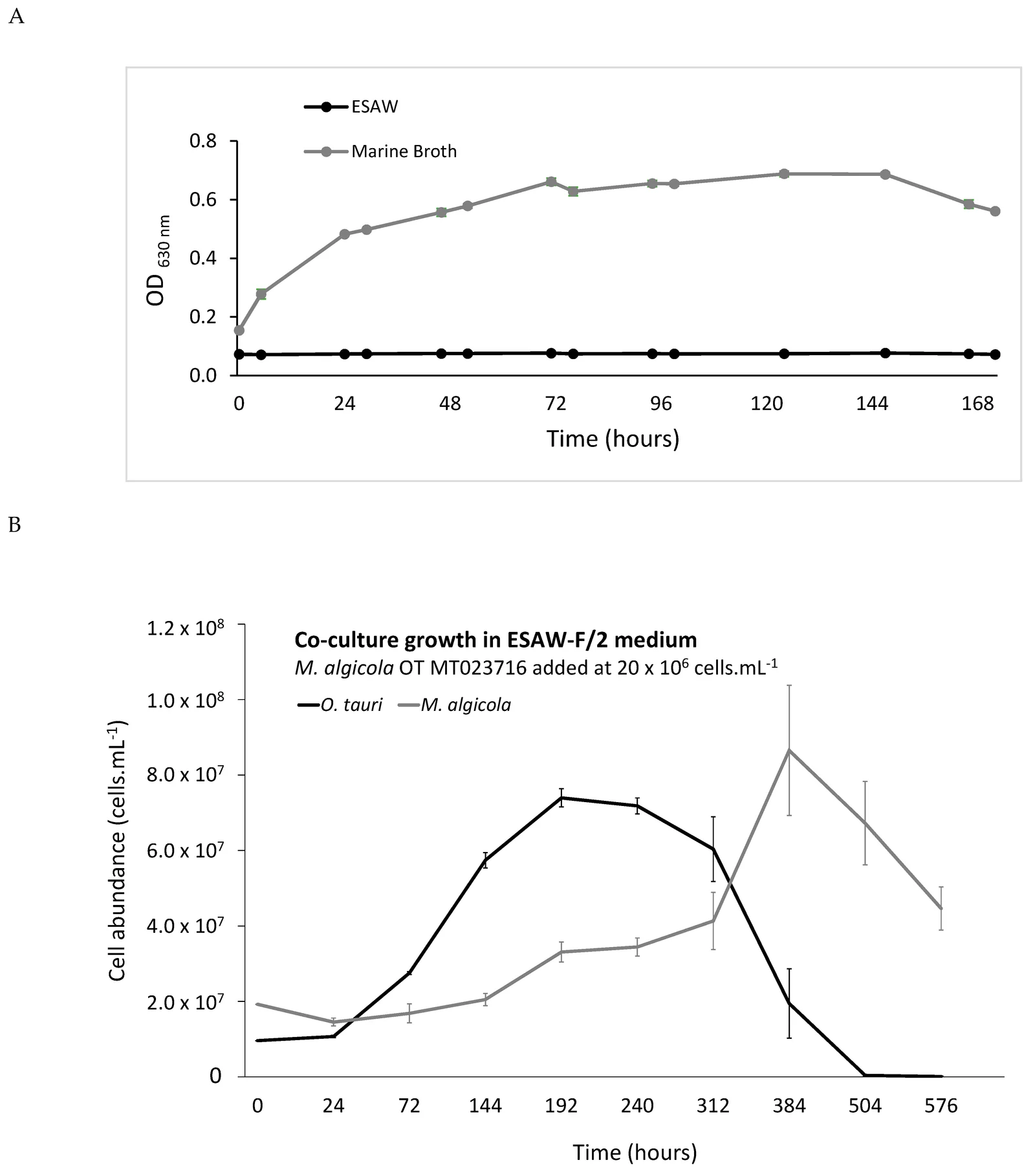
*M. algicola* OT MT023716 growth under different experimental conditions. (**A**) *M. algicola* OT MT023716 monoculture growth in ESAW-F/2 and marine broth media; (**B**) *O. tauri* RCC4221 and *M. algicola* OT MT023716 coculture growth in ESAW minus vitamin B_12_, showing that a sufficient number of *O. tauri* cells in stationary phase induces *M. algicola* OT MT023716 growth.

A bacterial monoculture experiment revealed that *M. algicola* OT did not grow in ESAW-F/2 medium contrary to what was observed in the positive control culture in MB medium (Figure 3A). However, *M. algicola* OT could grow in ESAW vitamin B_12_-depleted medium in coculture with *O. tauri* RCC4221 (ESAW minus B_12_, Figure 3B). *M. algicola* OT started to grow at day 8 and reached its maximal cell abundance at day 16, while *O. tauri* RCC4221 growth began at day 3 to reach maximal cell abundance at day 8 and then declined until complete cell disappearance at day 21 (Figure 3B). The *M. algicola* exponential growth phase was strongly delayed compared to the *O. tauri* one (10 days for *M. algicola* OT versus 1 day for *O. tauri*, Figure 3B). The two species exhibited shifted growth curves.

### 3.5. M. algicola OT Growth on Lipids

**Figure 4:**
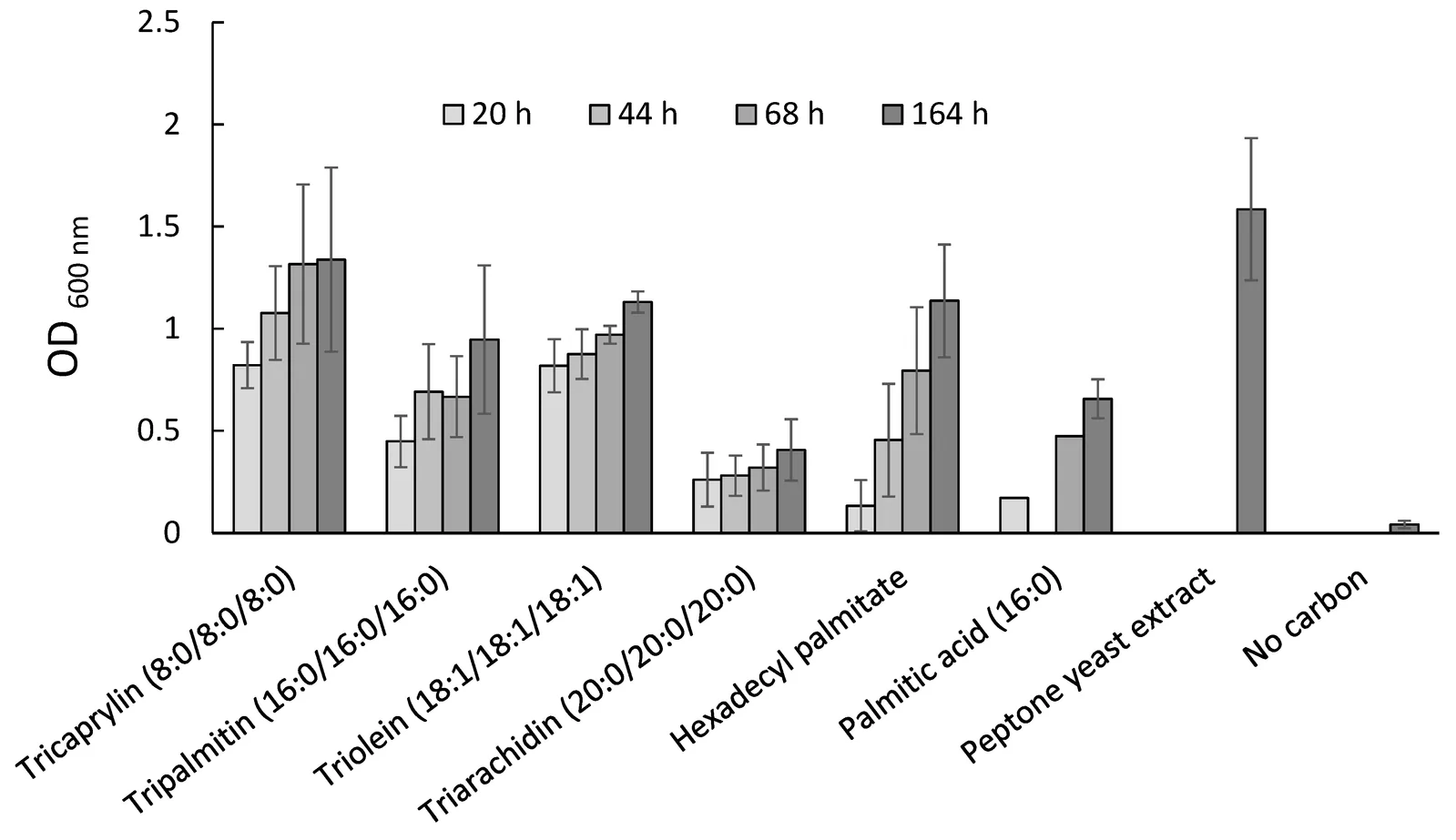
*M. algicola* OT MT023716 growth on lipids and other sources.

For each compound tested, the *M. algicola* OT growth (until OD = 1.34 for the highest, Figure 4) was obtained at four different tested times, ranging from 20 to 164 h (Figure 4). Figure 4 shows that *M. algicola OT* was able to grow on four TAGs (tricaprylin, tripalmitin, triolein and triarachidin), a wax ester (hexadecyl palmitate) and a fatty acid (palmitic acid). These results indicated that *M. algicola OT* was able to assimilate the tested lipids. However, it was not possible to draw any conclusions from these data regarding the efficiency of the assimilation of the different lipids as they were nearly insoluble in water and their assimilation rate depended on the area of the water-lipid interface. The ability of *M. algicola* OT to assimilate a variety of lipids was also suggested by the occurrence of lipids and degradation genes in the *M. algicola* DG893 genome reference (if transcribed and translated). The two genes *fadA* and *fadB* target genes coding for proteins involved in lipid degradation, were found with relatively high coverage in genomics data of *O. tauri* phycosphere cultures (Table 3). Complete screening of available *Marinobacter* genomes also revealed that these two genes were retrieved in the *M. algicola* DB893 genome reference (Table 2) (with *p*-values = 0 after BLASTP searching). The occurrence of *fadB* and *fadA* indicates the probable presence of a fatty acid oxidation pathway in *M. algicola* DG893, and thus probably in *M. algicola* OT.

### 3.6. Attempts to Detect AI-1 or AI-2 from M. algicola OT by Experimentation or Genomic Screening

We evaluated the production of AHLs by *M. algicola* OT using sensor strains. The *M. algicola* OT strain did not produce type 1 (Figure S1A) or type 2 (Figure S1B) auto-inducers. However, genes coding for proteins involved in QS, mainly *luxI*, *luxS* and *hdtS*, were present with relatively high coverage in *O. tauri* phycosphere cultures. These genes belong to taxonomically distinct bacteria (Table S1) and were all present in *O. tauri* RCC4221 culture at very low abundance rate [25]. However, complete screening of available *Marinobacter* genomes did not identify any homologues of the *luxI*, *luxS*, *luxM*, *vanM*, *ainS* or *hdtS* QS genes involved in AI synthesis. Nevertheless, LuxR receptor homologues were retrieved in 13 out of 21 *Marinobacter* genomes, including *M. algicola* DG893 (Table S2). INTERPRO and SMART analysis of the *Marinobacter* LuxR protein sequences confirmed that all of them harbour the typical LuxR DNA-binding helix-turn-helix (HTH) domain at their C-terminal region. In the N-terminal region, eight sequences presented a signal transduction response regulator domain (IPR001789), three presented a Malt-like TPR region (IPR041617) and two presented N terminal domains that were not affiliated with any known type of protein domain. However, none of them presented the AI-binding domain (IPR005143), which was confirmed by a COACH-based analysis and alignment of LuxR sequences with the *Vibrio fischeri* and *Agrobacterium* LuxR sequences (personnal observation).

## 4. Discussion

### 4.1. Contrasting Behaviours of Marinobacter Strains in Microalgae Cultures

Bacterial growth can be substantially favoured because of organic nutrients in the surrounding environment. During blooms in Mediterranean coast, Trombetta and collaborators noticed dominant positive relationships between bacteria and phytoplankton [7] and it is known that bacterial cells can satisfy part of their own carbon demand by consuming phytoplankton exudates [57,58]. In coculture with *O. tauri*, organic matter quantity strongly depends on the growth stage of the microalgae, being particularly low until the middle of the exponential phase and high once the *O. tauri* cells reach the stationary phase. From our results, we hypothesise that bacteria largely benefit from *O. tauri* dead cells directly or from the exudates of accumulating living cells during exponential and decline phases. The behaviours of *O. tauri* RCC4221 and *M. algicola* in coculture contrast. *Ostreococcus tauri* is a known B_12_ auxotroph [21]. The maximal cell abundance of *O. tauri* was 2.5-fold lower in the ESAW medium without vitamin B_12_ than in the ESAW-F/2 complete medium in the control cultures, thus confirming that *O. tauri* RCC4221 needs vitamin B_12_ for optimal growth. The presence of vitamin B_12_ biosynthetic genes in many *Marinobacter* species suggested that *M. algicola* OT could provide vitamin B_12_ to *O. tauri* RCC4221. However, here, we did not demonstrate any impact of *M. algicola* OT in favour of *O. tauri* RCC4221 growth in coculture when the culture medium was depleted in vitamin B_12_. From our experiments, *M. algicola* OT could not provide vitamin B_12_ to *O. tauri* RCC4221. In contrast, the decline in *O. tauri* cells obviously matched the beginning of *M. algicola* OT growth, arguing that bacteria largely benefit from *O. tauri* (directly from dead cells or from the exudates of accumulating living cells) to sustain their growth. However, *O. tauri* was maintained at the highest density from day 21 to the end of the experiment when the initial density of *M. algicola* OT was twice that of the microalgae. This finding could indicate that *O. tauri* RCC4221 survived longer in the presence of the bacteria, probably benefiting from some decomposition of organic matter during the decline phase. Nevertheless, in a recent study, another *Marinobacter* behaviour, one that favoured *O. tauri* growth in culture, was evidenced [59]. In that study, the occurrence of a *Marinobacter* sp. OTB1 strain was reported in cultures of the *O. tauri* RCC745 strain, phylogenetically close to our *O. tauri* RCC4221 strain. The authors showed that *Marinobacter* sp. OTB1 favours the *O. tauri* RCC745 growth. They showed that in presence of *Marinobacter* sp. OTB1, the growth of *O. tauri* was stimulated following the addition of a precursor component in vitamin B_1_-limited cultures. In contrast, no growth stimulation was observed when antibiotics were added to prevent bacterial growth [59], therefore demonstrating the beneficial presence of bacteria for microalgae growth. In our case, even in the presence of vitamin B_12_, the presence of *M. algicola* OT did not enhance *O. tauri* RCC4221 growth in culture. It can be a little paradoxical that phylogenetically closed *O. tauri* strains (RCC4221 and RCC745) harbouring *Marinobacter* strains in their laboratory culture phycospheres, are not impacted the same way depending on the *Marinobacter* strain. In a previous study, the growth of two *Prochlorococcus* strains (MIT9313 and MED4) was differentially affected when they were cultivated with the same collection of heterotrophic bacteria [60]. The authors highlighted that the kind of interaction was dependent on the phylogenetic signature. From this point of view, closely related bacteria (whose 16S gene sequences differed by 1–2%) show more similar effects in cultures [60]. As pointed out previously from our phylogenetic reconstruction, *M. algicola* OT and *Marinobacter* OTB1 are two independent species that are not closely related, showing 5.3% differences in a 16S partial alignment (corresponding to the length of the 16S partial sequence of *Marinobacter* OTB1), and thus probably exhibit different metabolisms in microalgae cultures. Last, in our phylogenetic reconstruction, the clustering of the green microalgae, dinoflagellate and haptophyte *Marinobacter* clade (*M. algicola*) and diatom-associated *Marinobacter* clade (*M. adhaerens*—*M. manganoxydans*—*M. flavimaris*) together with *M. salsuginis* provided a highly bootstrap support (BP = 83). Contrary to all *Marinobacter* species mentioned above, which include bacteria with neutral or positive effects on microalgae to our knowledge, the *M. salsuginis* BS2 strain is the only species that does not seem to exhibit positive behaviour towards microalgae. The *M. salsuginis* BS2 strain was discovered from environmental sediments only two years after *M. algicola* was discovered. Although it appears closely phylogenetically related to *M. algicola* in our phylogenetic reconstruction, the *M. salsuginis* BS2 is an algicidal bacterium showing killing activity towards the harmful bloom-forming dinoflagellate *Noctiluca scintillans* [10].

### 4.2. M. algicola OT Metabolises Different Lipids

The growth defect of *M. algicola* OT in ESAW-F/2 without *O. tauri* RCC4221 was most likely due to the absence of a carbon and energy source in this medium. *O. tauri* RCC4221 is known to produce a wide range of lipids including TAGs that represent approximately 15% of the total lipids under nutrient-replete conditions and that accumulate up to 10 times more in nitrogen-depleted conditions [35]. Many *Marinobacter* species are known to degrade a large variety of hydrophobic substrates, including TAGs, wax esters, fatty acids, fatty alcohols and alkanes [31,61]. The ability of *M. algicola* OT MT023716 to grow in the presence of *O. tauri* RCC4221 in ESAW-F/2 medium, while it did not grow alone, suggested that the bacterium benefited from some exudates of *O. tauri*. From our experiments, *M. algicola* OT grew in the presence of different lipids, including four different TAGs. We can hypothesise that *M. algicola OT* might take advantage of TAGs produced by *O. tauri*, which are some of its major lipids [35]. In *M. algicola* OT and *O. tauri* coculture experiments, the co-occurrence of the onsets of the growth of the bacterium with the stationary phase of the algae is consistent with a hypothetical release of lipids or other substrates by *O. tauri* during the stationary phase, possibly by cell lysis. Even if it does not reveal they are actively transcribed and translated, the presence of different genes involved in different fatty acid degradation pathways in *M. algicola* DG893 would be a strong indicator to corroborate this hypothesis. In addition, *M. algicola* OT might also degrade starch from the microalga if an alpha-amylase (glycosidase) present in the *M. algicola* DG893 genome (personal observation) was also actively synthesised. Further investigations will be needed to directly assess the growth of *M. algicola* OT on *O. tauri* lipidic extracts and determine which microalga lipids are particularly metabolised.

### 4.3. M. algicola OT Does Not Produce AI-1 or AI-2 QS Autoinducers

The capacity of *Marinobacter* species to communicate by QS should be an essential feature of the genus. *M. hydrocarbonoclasticus*, an oil-degrading bacterium, forms biofilms at the substrate–water interface [62], when carbon sources such as lipids are condensed in nonaqueous phases in marine waters. Producing biofilms is a particular bacterial activity that is commonly regulated by QS in numerous bacterial families. Our first global screening for genes coding for proteins involved in QS found many QS genes involved in AI synthesis in the global phycosphere of *O. tauri* RCC4221 laboratory cultures. However, *M. algicola* and all other *Marinobacter* species screened do not possess any of the classical QS genes. Contrary to what we expected given the high prevalence of *M. algicola* OT in *O. tauri* RCC4221 cultures, the *M. algicola* OT MT023716 strain did not produce either AI-1 or AI-2 according to our various detection tests. In contrast, a *Marinobacter* strain recovered from snow particles in a marine environment (*Marinobacter* sp. PCOB-2 HP36, AJ000647) produce AHLs [34]. In our phylogenetic reconstruction, *Marinobacter* sp. PCOB-2 was not related to the *M. algicola* clade but rather to the diatom-associated *Marinobacter* clade, which included *M. adhaerens* known to live closely attached to diatoms. However, our genomic screening found no evidence of the QS genes involved in AI-1 and AI-2 synthesis in any *Marinobacter* genome available, including *M. adhaerens*. Nevertheless, other types of QS modes are possible in Gram-negative bacteria, but their study requires complex and in-depth multidisciplinary studies that are out of the context of this current work. In addition, except in a study from Gram and coworkers [34], experimental evidence of QS capacity in any *Marinobacter* strain has never been demonstrated. False positives are regularly observed in this kind of experiment [63], and thus, we hypothesise that this could probably be the case in the Gram and coworkers study unless new experiments demonstrate otherwise. Meanwhile, the encoding of a Lux-R receptor (assuming it is correctly translated and functional) in the majority of the *Marinobacter* genomes available, including *M. algicola* DG893, suggests that *M. algicola* senses AI from other bacterial communities and benefits from them to synchronise its own activities. However, for the thirteen retrieved *M. algicola* LuxR protein sequences, *in silico* protein analyses revealed the lack of an AI-binding domain (and of other features such as conserved amino acids involved in AHL binding and the presence of InterPro domains) in the LuxR protein sequences, thus eliminating this kind of ‘cheater’ behaviour [64]. In our study, we more specifically focused on AI-1 (AHL-driven) and AI-2 (furanosyl diester borate-driven) modes of QS. However, other types of QS have been depicted in the literature [65]. As their study requires intense experimental work, we did not consider including them in the context of this study. Nevertheless, their inclusion in future research could provide interesting insights into *Marinobacter* physiology, especially in the context of microalgae-bacteria interactions [32].

### 4.4. M. algicola OT Is a Commensal Species in O. tauri Cultures and Is Widespread in the Natural Environment

It appears clear that the colonisation of different phycospheres by *Marinobacter* sp. strains under laboratory conditions not only occurs in dinoflagellate cultures in which it was initially described [56] but can be extended to coccolithophorid, diatom and Chlorophyta microalgae cultures, including cultures with *O. tauri* RCC4221. From our partial 18S phylogenetic reconstruction, these strains all cluster together. It seems that bacterial species that best behave throughout rapid host growth have been selected from the diversity of bacteria observed in phytoplanktonic cultures, as previously suggested [12]. We wonder if this kind of selection over generations is a general phenomenon that also extends to other microalgae cultures to result in *M. algicola* OT MT023716 predominance in *O. tauri* RCC4221 cultures. The *M. algicola* OT growth phase matched the decline in *O. tauri* cells, suggesting that the bacteria fed on the exudates or dead cells of the algae. A study by Trombetta and collaborators during bloom period in mediterranean coast [7] revealed evidence of positive interactions between phytoplankton and bacteria that suggest that vitamin-synthesising bacteria would provide vitamins to phytoplankton in exchange for organic carbon [19,66,67]. Our results show that without an adequate carbon source, *M. algicola* is difficult to cultivate and grows particularly well in phytoplanktonic cultures in which it probably finds additional nutrients provided by living and/or dead algal cells. Given the relatively low variability level inside the *M. algicola* clade, it is not possible to state if *M. algicola* strains found in dinoflagellate cultures are more closely related to each other than to the other *M. algicola* strains inside the clade, which would indicate whether the bacteria are specific to some kinds of microalgae. In addition, a recent study reported the occurrence of the *Marinobacter* sp. OTB1 strain [59] in cultures of *O. tauri* RCC745. Another recent study suggests that algal-associated bacterial communities are controlled by algal hosts, culture conditions and the initial inoculum composition of the microalgae phycosphere [68].

The range of lipid compounds allowing *M. algicola* growth that were highlighted in this study could be the key elements explaining the occurrence of this bacterial species in so many different microalgae laboratory cultures. Interestingly, the majority of *Marinobacter* strains found in diverse Chlorophyta, Dinoflagellates and Haptophyte cultures are all in the *M. algicola* clade in our phylogenetic reconstruction, demonstrating a particular acclimation of this opportunistic species in different phycospheres in laboratory cultures. In the presence of organic carbon, *Marinobacter* can grow rapidly, out-competing other bacteria in enrichment cultures [69] and easily dominates other communities in natural marine aggregates [70]. This r-strategist (or copiotrophic) behaviour renders them very easy to cultivate, even compared with other heterotrophic marine bacteria [71]. *Marinobacter* species probably use other kinds of communication system to optimise their growth, and these bacteria might form biofilms, a central mechanism in the natural environment [72], to colonise any substrates that are not well dissolved [61], including phytoplanktonic blooms. Preliminary exploration of public datasets from Tara Oceans reveals probable colocalisation of *M. algicola* and *O. tauri* in coastal environments. It is highly probable that *O. tauri*, as a bloom-forming phytoplanktonic species, can be an important nutritive interface for numerous bacterial strains in the natural environment.

## 5. Conclusions

The persistence of *M. algicola* OT MT023716 bacteria in *O. tauri* RCC4221 cultures would result from opportunistic behaviour. Our experiments suggest that the *M. algicola* OT MT023716 strain needs *O. tauri* microalgae to grow in synthetic medium. Although neither genomic evidence nor experimental work argued in favour of QS activity by *M. algicola* OT MT023716, the strain grows at high density on a wide range of lipids, including triglycerides, known to be produced by *O. tauri* and numerous marine microalgae in culture during abiotic stress. *Marinobacter algicola* strains are frequently found in different microalgae cultures in the laboratory, which reveals their plasticity in colonising different laboratory culture phycospheres. Understanding why this colonisation is limited to certain phytoplanktonic species in the laboratory will bring new insights into the optimum activity of *M. algicola* in cultures, and perhaps also in marine environments.

## Figures and Tables

**Table 1:** BWA alignment results of vitamin B_12_ synthase genes with raw genomics reads from 19 *O. tauri* (and its phycosphere) line cultures. From these 19 independent lines, which were endpoints diluted through serial subcultures [41], all the cultivable bacteria in solid media were determined to be *M. algicola* OT MT023716 [25].

B_12_ Vitamin Synthase Genes	Putative Organism Origin	NCBI Accession Number
*metH* *Methionine synthase*	*Marinobacter algicola*	NZ_ABCP01000002
*metE* *Methionine synthase*	*Marinobacter algicola*	NZ_ABCP01000027

**Table 2:** Screening of methionine synthase genes and lipid degradation genes in 21 *Marinobacter* complete genomes (accession numbers in the NCBI). Vitamin B_12_ synthase genes are methionine synthase genes (*metH* and *metE*). Lipid degradation genes are fatty acid degradation alpha subunit genes (*fadB*) and 3-ketoacyl-CoA thiolase genes (*fadA*).

*Marinobacter* Strains	Accession Number	*metH*	*metE*	*fadB*	*fadA*
*M. adhaerens* HP15	PRJNA224116	**√**	**√**	**√**	**√**
*M. algicola* DG893	PRJNA19321	**√**	**√**	**√**	**√**
*M. antarcticus* CGMCC 1.10835	PRJEB18348	**√**		**√**	**√**
*M. daepoensis* DSM 16072	PRJNA195792	**√**	**√**	**√**	**√**
*M. excellens* HL-55	PRJNA195885	**√**	**√**	**√**	**√**
*M. hydrocarbonoclasticus* ATCC49840	PRJEA91119	**√**	**√**	**√**	**√**
*M. lipolyticus* SM19	PRJNA196694	**√**	**√**	**√**	**√**
*M. lutaoensis* T5054	PRJNA357186	**√**	**√**	**√**	**√**
*M. manganoxydans* MnI7-9	PRJNA73991	**√**	**√**	**√**	**√**
*M. mobilis* CGMCC 1.7059	PRJEB16583	**√**	**√**	**√**	**√**
*M. nanhaiticus* D15-8W	PRJNA193181	**√**		**√**	**√**
*M. nitratireducens* AK21	PRJNA178951	**√**	**√**	**√**	**√**
*M. pelagius* CGMCC 1.6775	PRJEB17496	**√**	**√**	**√**	**√**
*M. persicus* IBRC-M 10445	PRJEB17403	**√**	**√**	**√**	**√**
*M. psychrophilus* 20041	PRJNA284323	**√**	**√**	**√**	**√**
*M. salarius* R9SW1	PRJNA227392			**√**	**√**
*M. salinus* Hb8	PRJNA349097	**√**	**√**	**√**	**√**
*M. santoriniensis* NKSG1	PRJNA188242	**√**	**√**	**√**	**√**
*M. segnicrescens* CGMCC 1.6489	PRJEB17056	**√**	**√**	**√**	**√**
*M. subterrani* JG233	PRJNA284629	**√**	**√**	**√**	**√**
*M. zhejiangensis* CGMCC 1.7061	PRJEB17480	**√**	**√**	**√**	**√**

**Table 3:** BWA alignment results of lipid degradation genes with raw genomics reads from 19 *O. tauri* (and its phycosphere) line cultures. From these 19 independent lines, which were endpoints diluted through serial subcultures [41], all the cultivable bacteria in solid media were determined to be *M. algicola* OT MT023716 [25]. Here, the most abundant lipid degradation genes found from the genomic screening of the culture data are presented.

Most Abundant Lipid Degradation Genes	Putative Organism Origin	Uniprot Accession Number
*fadB* *Fatty acid degradation alpha subunit*	*Pseudomonas putida*	F8FUP1
*fadA* *3-ketoacyl-CoA thiolase*	*Pseudomonas putida*	F8FUP2

## Data Availability

Not applicable.

## Supplementary Materials

The following are available online at https://www.mdpi.com/article/10.3390/microorganisms9081777/s1, Figure S1: No AI-1 (Figure S1A) or AI-2 (Figure S1B) production by *M. algicola* OT, Table S1: BWA alignment results of reference QS genes with raw genomics reads from 19 *O. tauri* (and its phycosphere) line cultures. Table S2: Quorum-sensing genes screening in 21 *Marinobacter* complete genomes (accession numbers in the NCBI). Text S1: Material and Methods for the evaluation of *M. algicola* OT quorum-sensing capacities.
