# Supplementary material for: Features of the Opportunistic Behaviour of the Marine Bacterium Marinobacter algicola in the Microalga Ostreococcus tauri Phycosphere
Source: Microorganisms. 2021 Aug 20;9(8):1777. doi: 10.3390/microorganisms9081777 (PMC8399681; doi:10.3390/microorganisms9081777)
Supplement: Supplementary file 1 [file microorganisms-09-01777-s001.zip › microorganisms-1327239-supplementary.pdf]

Supplementary Material

Text S1

Material and Methods for the distinction of *M. algicola* OT from other bacterial communities in *O. tauri* RCC4221 cultures.

O. tauri RCC4221 cultures used for coculture experiments were not axenic despite the use of antibiotic treatments. At the lowest bacterial contamination (around 3%), *O. tauri* RCC4221 culture was diluted to 10^6 cells.mL^{-1} in F/2-ESAW medium and distributed into several 50 mL sterile flasks. Co-culture experiments were performed for a total duration of 30 days. To test the effects of *M. algicola* OT on *O. tauri* RCC4221, different final bacterial concentrations (5×10^6 and 20×10^6 *M. algicola* OT cells.mL^{-1}) were added to *O. tauri* culture RCC4221. A control (without the addition of *M. algicola* OT to the *O. tauri* RCC4221 culture) allowed us to visualize the growth pattern of the few remaining bacteria (fewer than 3×10^5 cells.mL^{-1}) after axenization was not totally efficient. *M. algicola* OT cells were distinguished easily from other bacterial communities in *O. tauri* RCC4221 cultures thanks to the former having weaker fluorescence signals after SYBR Green labelling than the other bacteria.

Text S2

Material and Methods for the evaluation of *M. algicola* OT quorum-sensing capacities

For the detection of AHLs:

E. coli MT102 and *P. putida* F117 were cultivated overnight with continuous shaking (200 rpm) at 37 °C in lysogeny broth (LB) medium developed by Bertani (Sigma L3022) and supplemented with tetracycline (25 µL.mL^{-1}) and at 30 °C in LB

supplemented with gentamicin (20 µL.mL^{-1}), respectively. An overnight culture of each biosensor strain was inoculated in fresh LB medium (dilution 1/50) and dispensed into 96-well microplates (150 µL.well^{-1} for bacterial supernatant evaluation and 180 µL.well^{-1} for extracts or fractions). To evaluate the ability of *M. algicola* OT to produce AI-1, culture supernatants were collected after centrifugation (10 min at 10,000 rpm) and added to microplate wells containing the biosensor strains (50 µL.well^{-1}, in triplicate). Microplates were incubated without shaking at 30 °C and 37 °C, depending on the optimum growth temperature of the selected biosensor strain. After 0, 5 and 24 h of incubation, fluorescence was determined with a Victor1420 Multilabel Counter (Perkin-Elmer) at an excitation wavelength of 485 nm and a detection wavelength of 535 nm. The optical density (OD) at 620 nm was also measured to monitor biosensor cell growth. Negative controls were biosensor cultures with sterile LB medium and without extract. Biosensor cultures with added commercial AHLs (C6-HSL for *E. coli* MT102 and oxo-C10-HSL for *P. putida* F117) were used as positive controls.

For the detection of AI-2:

Briefly, a *V. harveyi* reporter strain was grown at 30 °C in autoinducer bioassay (AB) medium. The overnight culture was then diluted 1:5,000 into fresh medium, and 180 µL of the diluted cells were added to wells containing 20 µL of the culture supernatants of the bacteria to be tested for AI-2 production. The 96-well plates were incubated at 30 °C. Every hour, luminescence was measured using a Victor1420 Multilabel Counter (Perkin-Elmer) until the medium control sample produced the least amount of light. AI-2 production is reported as the fold induction of the reporter strain divided by the background when only medium was added to the reporter. All assays were performed in duplicate.

Protocols are referenced in Tourneroche et al. (2019) and Doberva et al. (2017)

Doberva M, Stien D, Sorres J, Hue N, Sanchez-Ferandin S, Eparvier V, Ferandin Y, Lebaron P, Lami R (2017) Large diversity and original structures of acyl-homoserine lactones in strain MOLA 401, a marine *Rhodobacteraceae* bacterium. Frontiers in Microbiology 8:1152

Tourneroche A, Lami R, Hubas C, Blanchet E, Vallet M, Escoubeyrou K, Paris A, Prado S (2019) Bacterial-fungal interactions in the kelp endomicrobiota drive autoinducer-2 quorum sensing. Frontiers in Microbiology 10:1693.

Figure S1

A

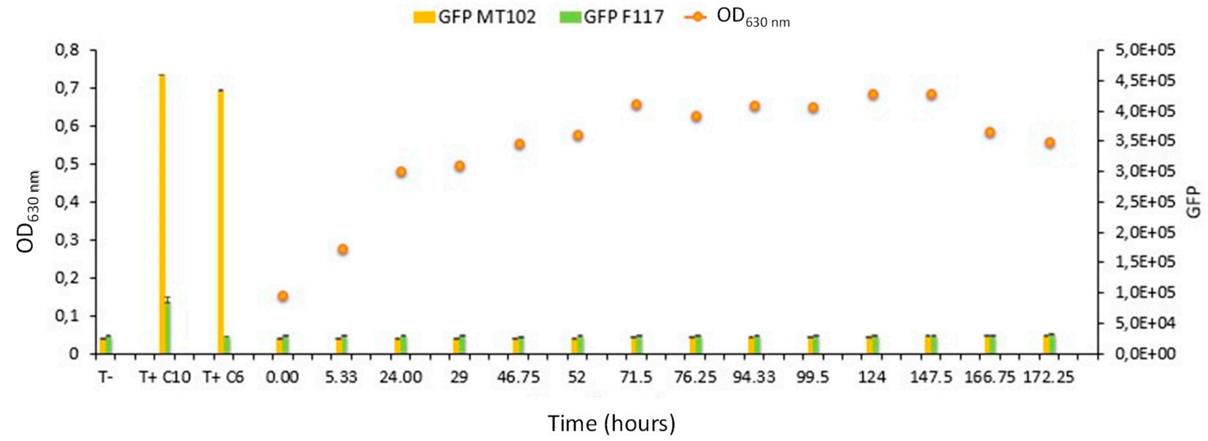

B

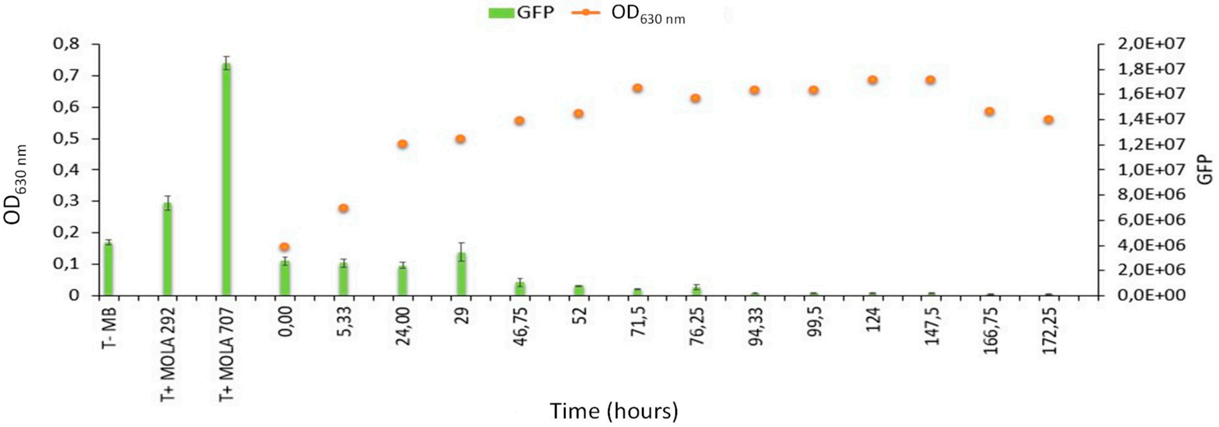

Figure S1: No AI-1 (Figure S2A) or AI-2 (Figure S2B) production by *M. algicola* OT. (A) AI-1 detection test of short chains by the *E. coli* MT102 biosensor (GFP MT102) and of long chains by P. putida F117 (GFP F117) during *M. algicola* growth (T- = marine broth only; T+C10 and T+C6 = supernatants from *Roseovarius tolerans* culture). The T + C10 and T + C6 control results show that the P. putida strain F117 specifically detects the long-chain, 10-carbon (T + C10) AI-1 and that *E. coli* MT102 detects the AI-1 long and short chains. For the negative control (T-) and the control T + C6 (chain containing 6 carbons, short chain) AI-1, the expression levels of GFP were not significantly different. The *M. algicola* strain does not produce type 1 AIs, regardless of whether they have long or short chains. The emission intensity of GFP

was equal to that of the negative control (T-) for the two biosensors used. (B) AI-2 detection test by the *Vibrio harveyi* MM32 biosensor along *M. algicola* (T- = marine broth only; T+ MOLA 292 and T+ MOLA 707 = supernatants from the MOLA station. The results also show that the *M. algicola* OT strain does not produce type 2 AIs. However, we noticed that the production of AI-2 differed with the time of growth, which did not occur in the two previous experiments.

Table S1: BWA alignment results of reference QS genes with raw genomics reads from 19 *O. tauri* (and its phycosphere) line cultures. From these 19 independent lines which were endpoints diluted through serial subcultures [41], all the cultivable bacteria in solid media were *M. algicola* [25]. Here are presented the 24 most abundant quorum-sensing genes in the cultures. No gene from *Marinobacter* species was found.

Most abundant quorum-sensing genes	Putative organism origin	Uniprot accession number
luxI	*Burkholderia multivorans*	AAG61138
luxI	*Sphingobium japonicum*	BAI95305
luxS	*Vibrionales* bacterium	EDK27865
luxI	*Burkholderia oklahomensis*	WP_010119585
luxI	*Burkholderia thailandensis*	YP439001
luxI	*Methylobacterium extorquens*	ABI74777
luxI	*Burkholderia pseudomallei*	YP_111576
luxI	*Ralstonia syzygii*	CCA85665
luxI	*Burkholderia vietnamiensis*	ACP43465
luxI	*Burkholderia mallei*	YP_106161
hdtS	*Bradyrhizobium* sp.	WP_009025797
luxI	*Burkholderia pseudomallei*	YP_111576
luxI	*Caulobacter* sp.	WP007662486
luxI	*Mesorhizobium alhagi*	WP008838483
hdtS	*Pseudomonas aeruginosa*	ZP04931075
hdtS	*Alcaligenes faecalis*	WP_003803484
luxI	*Ruegeria conchae*	WP_010442847
hdtS	*Pseudomonas synxantha*	EIK72202
luxI	*Burkholderia multivorans*	EJO60396
hdtS	*Pseudomonas psychrotolerans*	ADG45384
hdtS	*Teredinibacter turnerae*	WP_018416032
hdtS	*Phaeospirillum molischianum*	WP_002725490
luxI	*Erwinia tracheiphila*	WP_016192680
luxI	*Collinsella_aerofaciens*	EBA38517

Table S2: Quorum-sensing genes screening in 21 *Marinobacter* complete genomes (accession numbers in the NCBI). Only *luxR* is present with high e-value in the annotation of some genomes for which the blast hit was "1-acyl-sn-glycerol-3-phosphate acyltransferase" (bold sign). *hdtS* was present is a lesser extent with not strong e-value (normal sign).

Marinobacter species	Accession number	*luxI*	*luxR*	*luxS*	*luxM*	*vanM*	*ainS*	*hdtS*
M. adhaerens HP15	PRJNA224116	-	√	-	-	-	-	-
M. algicola DG893	PRJNA19321	-	√	-	-	-	-	-
M. antarcticus CGMCC 1.10835	PRJEB18348	-	-	-	-	-	-	-
M. daepoensis DSM 16072	PRJNA195792	-	√	-	-	-	-	-
M. excellens HL-55	PRJNA195885	-	√	-	-	-	-	-
M. hydrocarbonoclasticus ATCC49840	PRJEA91119	-	√	-	-	-	-	√
M. lipolyticus SM19	PRJNA196694	-	√	-	-	-	-	√
M. lutaoensis T5054	PRJNA357186	-	-	-	-	-	-	√
M. manganoxydans MnI7-9	PRJNA73991	-	√	-	-	-	-	√
M. mobilis CGMCC 1.7059	PRJEB16583	-		-	-	-	-	√
M. nanhaiticus D15-8W	PRJNA193181	-	√	-	-	-	-	√
M. nitratireducens AK21	PRJNA178951	-	√	-	-	-	-	-
M. pelagius CGMCC 1.6775	PRJEB17496	-	-	-	-	-	-	√
M. persicus IBRC-M 10445	PRJEB17403	-	-	-	-	-	-	-
M. psychrophilus 20041	PRJNA284323	-	-	-	-	-	-	-
M. salarius R9SW1	PRJNA227392	-	√	-	-	-	-	-
M. salinus Hb8	PRJNA349097	-	√	-	-	-	-	√
M. santoriniensis NKSG1	PRJNA188242	-	-	-	-	-	-	√
M. segnicrescens CGMCC 1.6489	PRJEB17056	-	√	-	-	-	-	-
M. subterrani JG233	PRJNA284629	-	√	-	-	-	-	√
M. zhejiangensis CGMCC 1.7061	PRJEB17480	-	-	-	-	-	-	-
